# Caruncle dysgeneses - A case series

**DOI:** 10.1016/j.ajoc.2023.101868

**Published:** 2023-06-10

**Authors:** Cornelius Jakob Wiedenmann, Thomas Reinhard, Claudia Auw-Hädrich

**Affiliations:** Eye Center, Medical Center, Faculty of Medicine, University of Freiburg, Freiburg im Breisgau, Germany

**Keywords:** Dysgeneses, Conjunctiva, Ocular adnexa, Caruncle tumour, Ophthalmic pathology

## Abstract

**Purpose:**

Caruncle dysgeneses are extremely rare and must be differentiated from caruncular and conjunctival tumors. Very few case reports with histopathological descriptions exist. In this case series, four patients with five caruncle dysgeneses, two with histopathological findings, are characterised.

**Observations:**

Patient 1, a 26-year-old woman, presented with a conjunctival change at the left lower eyelid she had first noticed seven months earlier. She reported foreign body sensation and itching. On her left eye was a subtarsal conjunctival tumour measuring approximately 4 × 4 mm with whitish sebaceous gland-like inclusions located almost in the fornix morphologically resembling the nearby caruncle. The patient was asymptomatic after excision. Histopathological examination of the excised tissue showed non-keratinizing squamous epithelium with goblet cells. Subepithelially, there was lymphoplasmacytic cellular infiltration with intervening epidermal cysts adjacent to sebaceous glands and underlying adipose tissue, but no hair follicles or sweat/lacrimal glands. The epidermal cysts contained scattered hairs. A diagnosis of supernumerary caruncle was made.

Patient 2, a 56-year-old woman, was referred for evaluation of a caruncle tumour that was reported to be present since childhood. Clinically the 5 × 5 mm measuring tumour appeared yellowish and less reflective compared to the normal caruncle tissue. Histopathologically, non-keratinizing squamous epithelium with goblet cells was found. In the area of more exposed tumour tissue, there were significantly fewer goblet cells and incipient keratinization of the superficial epithelial layers. Subepithelially, sebaceous glands and adipocytes were present. Hair follicles or sweat/lacrimal glands were not evident. A diagnosis of megacaruncle was made.

Patient 3, a 58-year-old woman with Goldenhar syndrome, was clinically diagnosed with a supernumerary caruncle on the right eye as an incidental finding.

Patient 4, a 24-year old man, clinically presented with a megacaruncle on the right eye and a supernumerary caruncle on the left.

**Conclusions:**

Caruncle dysgeneses are often asymptomatic and have to be differentiated from other caruncular and conjunctival tumors. If they are present, attention should be paid to signs of an oculo-auriculo-vertebral spectrum as Goldenhar syndrome. In case of unclear findings or complaints, excision with subsequent histopathological examination is required.

## 1 Introduction

The caruncle is a small pinkish tissue hump in the nasal corner of the eyelid. It lies adjacent to the acute-angled fusion of the upper and lower eyelids. Laterally, the caruncle is bordered by the plica semilunaris. The caruncle consists of conjunctiva, hair follicles, sebaceous glands, and accessory lacrimal glands. Caruncle tumors, neoplastic and non-neoplastic, have already been described.^1^, ^2^, ^3^, ^4^, ^5^ However, except for a few case reports, often missing histopathological examination, much less is known about dysgeneses of the caruncle.^6^, ^7^, ^8^ For dysgeneses of the caruncle, a classification into supernumerary caruncles, ectopic caruncles, megacaruncles, and bilateral dysplastic caruncles has been proposed.^6^ Although caruncle dysgeneses are often asymptomatic and occur in otherwise healthy individuals, they are common in Goldenhar syndrome,^9^ which is characterized by malformations of structures derived from the first and second branchial arches, including the eyes, lips, tongue, palate, ear, maxilla and mandible. In this case series, we report on four patients with dysgeneses of the caruncle, two of which were histologically confirmed. One patient had a diagnosis of Goldenhar syndrome.

A keyword search was performed in the database at the Department of Ophthalmology, University Hospital Freiburg, using the word “caruncle” from January 1, 2006, to May 19, 2022. Where available, all written and photographic documents were examined for evidence of caruncle dysgeneses. The localization, subjective complaints, histological characteristics, and measures performed were taken from the records. Additionally, we conducted a literature research via MEDLINE with the search terms “supernumerary caruncle”, “ectopic caruncle”, “megacaruncle”, “lacrimal caruncle dysgenesis” and “caruncle malformation”. Written informed consent for publication of their clinical details and clinical images was obtained from the patients. Approval was given by the local Ethics Committee of the University of Freiburg under the following number (22/15).

## 2 Findings

### 2.1 Case 1

#### 2.1.1 Anamnesis

A 26-year-old female patient was referred to the Eye Center for co-evaluation of a conjunctival tumor and suspected pyogenic granuloma on the tarsal lower lid of the left eye (LE). The patient reported that the conjunctival change was first noticed seven months earlier and was initially treated with lubricating eye drops. Subjectively, the findings had been stable in size during the last seven months. There was no bleeding. The patient suffered from foreign body sensation and itching at the LE. Currently, there was no ophthalmologic therapy.

#### 2.1.2 Clinical findings and therapy

**Fig. 1:**
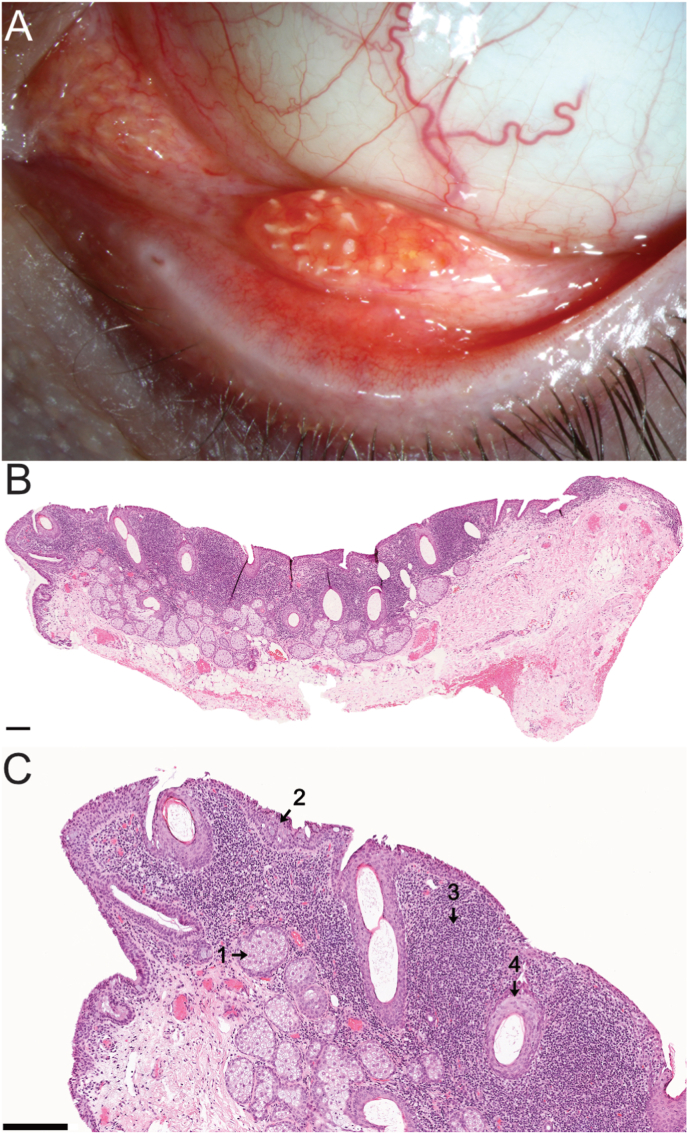
Clinical photograph and histopathology of patient 1. A: Clinical photograph of patient 1. The clinical image shows a tumor in the lower fornix. The aspect resembles the caruncle (see caruncle on the left side of the image). Sebaceous glands with its orifices are seen at the surface of the lesion. In comparison to the caruncle, the tumor is more reddish. There is no direct connection of the tumor to the caruncle. B: Histopathological overview of supernumerary caruncle. HE-staining. Bar equals 200 μm. C: Histopathological details of supernumerary caruncle. 1. Sebaceous glands, 2. Goblet cells, 3. Lymphoplasmacellular infiltration, 4. Epidermal cysts. HE-staining. Bar equals 200 μm.

Best corrected visual acuity (BCVA) on both eyes (BE) was 20/20 (right eye (RE) +0,5/−1,0/178°, LE +0,25/−0,25/178°). Intraocular pressure (IOP) was normotensive at 16 mm Hg bilaterally. The RE showed no abnormalities of the conjunctiva or the anterior segment except for subtarsal follicles. On the LE, there was a subtarsal conjunctival tumor measuring approximately 4 × 4 mm with whitish sebaceous gland-like inclusions located almost in the fornix, which morphologically resembled the nearby caruncle (Fig. 1A). However, compared to the caruncle, the subtarsal tumor was slightly more reddish and sat broad-based slightly elevated on the conjunctiva. There were no hairs in the area of the tumor.

With subjective complaints at the LE and atypical findings for a pyogenic granuloma, we decided together with the patient to excise the conjunctival tumor. Subsequently, a therapy with ofloxacin eye ointment three times daily for five days was recommended. Four weeks postoperatively, the patient reported a decrease of the previously described symptoms.

#### 2.1.3 Histologic findings

Histopathological examination of the excised tissue was performed by haematoxylin-eosin (HE) staining (Fig. 1B&C) and periodic acid-Schiff (PAS) reaction. Epithelium composed of nonkeratinizing squamous epithelium that was four to six cells thick with an admixture of goblet cells. Subepithelially, there was marked lymphoplasmacytic cellular infiltration with intervening epidermal cysts adjacent to sebaceous glands and underlying adipose tissue. Hair follicles or sweat/lacrimal glands were not found. The epidermal cysts contained scattered hairs. A supernumerary caruncle of the LE was diagnosed.

### 2.2 Case 2

#### 2.2.1 Anamnesis

A 56-year-old female patient was referred for evaluation of a caruncle tumor on the nasal corner of the lid of the LE. The patient reported that the change had been present since childhood and had grown over the past one to two years. Subjectively, the patient did not feel affected by the tumor on her LE.

#### 2.2.2 Clinical findings and therapy

**Fig. 2:**
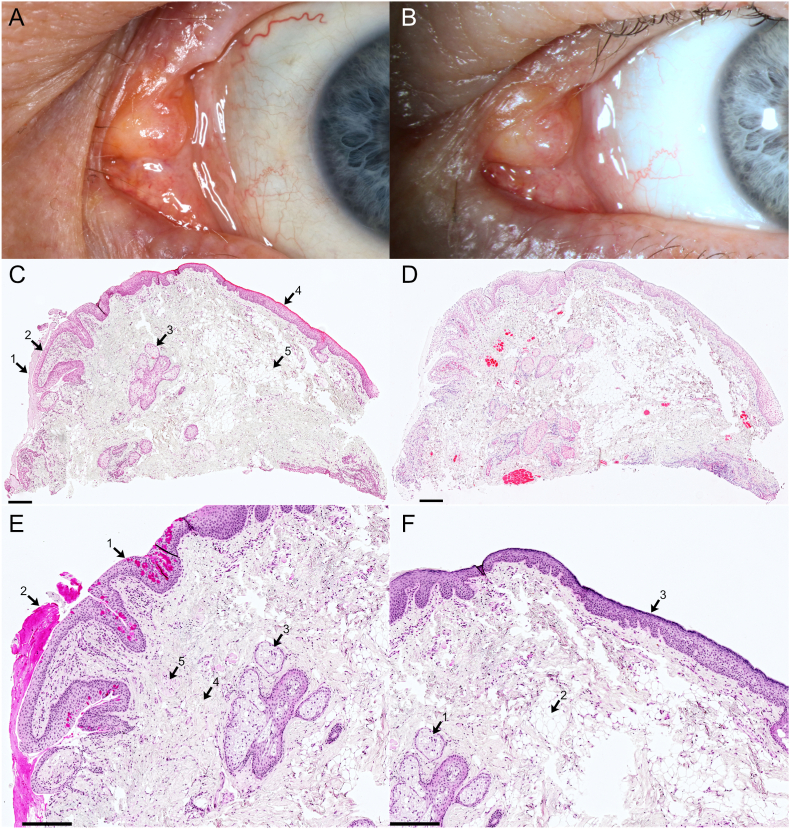
Clinical photograph and histopathology of patient 2. **A&B:** Clinical photograph of patient 2. Caruncle tumor at first presentation (A) and two years later (B). Above and directly adjacent to the caruncle, a slightly yellowish conjunctival tumor is obvious. The surface in the area of the tumor appears slightly dull as a possible sign of keratinization. There is no hemorrhage, ulceration or telangiectasia. The findings remained largely unchanged over the course of two years. **C–F:** Histopathological image of megacaruncle. C: 1. Mucus, 2. Non-keratinizing squamous epithelium with goblet cells, 3. Sebaceous glands, 4. Squamous epithelium with beginning keratinization, 5. Adipocytes. HE-staining. Bar equals 250 μm. D: Immunohistology for Actin. Red stained areas represent Actin-positive areas, which are most likely attributable to myocytes. Bar equals 250 μm. E: Magnification of the left upper part of C: 1. Non-keratinizing squamous epithelium with goblet cells, 2. Mucus, 3. Sebaceous glands, 4. Connective tissue, 5. Muscle fibers. PAS-staining. Bar equals 200 μm. F: Magnification of the right upper part of C: 1. Sebaceous glands, 2. Adipocytes, 3. Squamous epithelium with beginning keratinization, PAS-staining. Bar equals 200 μm. (For interpretation of the references to colour in this figure legend, the reader is referred to the Web version of this article.)

BCVA on the RE was 20/20 (+1,0/−1,25/3°) and on the LE 20/25 (−0,25/−0,75/154°). IOP was normotensive on BE (18/19 mm Hg). The RE showed no abnormalities of the conjunctiva, caruncle, or anterior segment except for a prolapse of fatty tissue. The LE showed a caruncle tumor adjacent to the normal caruncle that appeared to be fused to the medial lid ligament (Fig. 2A&B). On slit lamp microscopy the tumor presented yellowish and less reflective compared to the normal caruncle tissue. The remaining ophthalmologic examination was normal. Due to personal circumstances, the patient did not reappear until two years later. Despite the stable findings after the two-year period, we decided together with the patient for an excision. The caruncle tumor of approx. 5 × 5 mm was excised and the patient was recommended a therapy with ofloxacin eye ointment three times daily for five days.

#### 2.2.3 Histopathological findings

In the histopathological examination, HE staining and PAS reaction revealed non-keratinizing squamous epithelium that was four to six cells thick and had an admixture of goblet cells (Fig. 2C–E). In the area of more exposed tumor tissue, there were significantly fewer goblet cells and incipient keratinization of the superficial epithelial layers. Subepithelially, sebaceous glands and adipocytes were present, as well as isolated myocytes that were positive for actin (Fig. 2F) but negative for desmin in immunohistochemistry. Hair follicles or sweat/lacrimal glands were not evident. A diagnosis of megacaruncle was made because the caruncle was enlarged overall and keratinization occurred only in the more exposed areas of the caruncle, indicating a reactive keratinization. Thus we consider the diagnosis of megacaruncle more likely than a dermoid.

### 2.3 Case 3

**Fig. 3:**
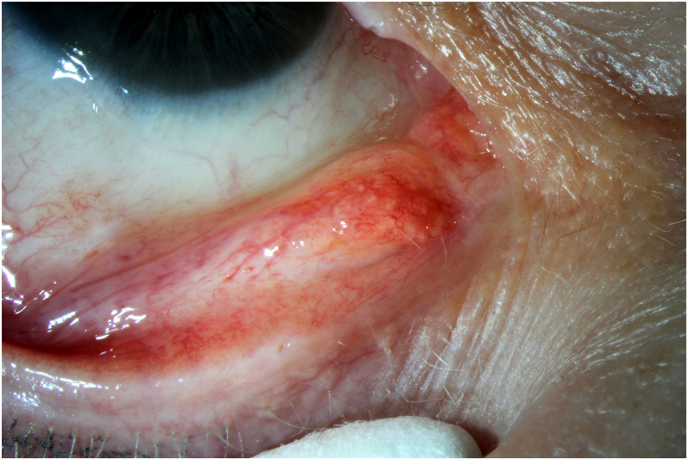
Clinical photograph of patient 3. A caruncle-like tumor lateral to the orthotopic caruncle (medial lid angle). Sebaceous glands and fine lanugo hairs are evident. No connection was found of the tumor to the caruncle.

A 58-year-old female patient with known Goldenhar syndrome presented. BCVA was 20/30 (+1.5/−0.75/46°) on the RE and 20/20 (+0.5/−0.75/14°) on the LE. She was diagnosed with a supernumerary caruncle on the RE as an incidental finding (Fig. 3). In addition, slit-lamp microscopy revealed findings consistent with bilateral epibulbar lipodermoid, and the findings were confirmed histologically after excision. The caruncle of BE was normal. Excision of the supernumerary caruncle was waived in the absence of symptoms and no signs of malignancy.

### 2.4 Case 4

**Fig. 4:**
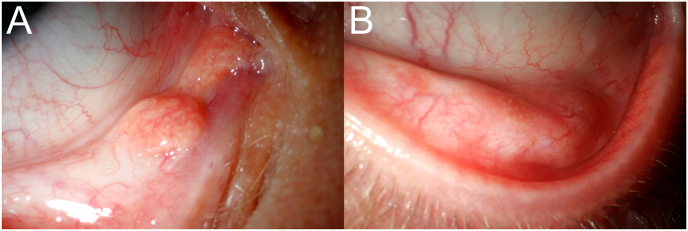
Clinical photograph of patient 4. A: Clinical photograph of patient 4. RE: The photograph shows a caruncle-like tumor with sebaceous glands and lanugo-hairs temporal in continuation to the caruncle. **B:** Clinical photograph of patient 4. LE: The photograph shows a flat caruncle-like tumor with sebaceous glands and lanugo-hairs temporal to the caruncle. No connection was found between the tumor and the caruncle.

A 24-year-old patient presented because he had noticed a change on both lower eyelids (Fig. 4) without any symptoms. Visual acuity was 20/20 on BE without correction. There were caruncle-like structures with sebaceous glands and lanugo hairs, more pronounced on the right side. We diagnosed a megacaruncle on the right and a supernumerary caruncle on the left eye. A check-up six months later revealed unchanged findings on both sides. Excision was waived in the absence of symptoms and no signs of malignancy.

### 2.5 General overview of all cases

Four cases of caruncle dysgeneses are presented. At the time of diagnosis of caruncle dysgeneses, all four patients were adults. The ratio of female to male patients was 3:1. Two cases involved the LE, one the RE, and one both eyes. One patient with a supernumerary caruncle reported foreign body sensation and itching. All other patients were asymptomatic. Three times a diagnosis of supernumerary caruncle and two times a diagnosis of megacaruncle was made. One patient with a supernumerary caruncle had an association with Goldenhar syndrome (Table 1).

**Table 1:** Characteristics of patients with caruncle dysgeneses.

Patient	Age	Gender	Laterality	Symptoms	Diagnosis	Goldenhar syndrome
1	26	f	LE	Foreign body sensation, itching	Supernumerary caruncle	no
2	56	f	LE	no	Megacaruncle	no
3	58	f	RE	no	Supernumerary caruncle	yes
4A	24	m	RE	no	Megacaruncle	no
4B	24	m	LE	no	Supernumerary caruncle	no

## 3 Discussion

In the present case series, four cases of the rare caruncle dysgeneses are described.

The exact embryological development of the caruncle has not been conclusively clarified. From the shiny surface of the orthotopic caruncle, 15 to 20 small lobules of sebaceous glands shimmer through the conjunctiva and fine, almost transparent lanugo hairs grow from hair follicles. Histopathologically, the caruncle-plica complex is covered by a goblet cell-rich, nonkeratinizing squamous epithelium. In addition to the hair follicle-gland units, PAS-positive lobules of accessory lacrimal glands may be evident. In a review by Jakobiec et al. several dysgeneses of the caruncle are described, which have to be distinguished from each other^6^:

1. The **supernumerary caruncle** is a rare congenital anomaly. In addition to the normal caruncle, additional tissue unrelated to the normal caruncle is present, showing histologic features similar to those of the latter. It may occur on the lower or upper medial third of the eyelid and may be single or multiple.

Given the normal caruncles on both eyes and the distinction of the caruncle from the tumor tissue by normal conjunctival tissue, our first case is most likely a supernumerary caruncle, even though no hair follicles and lacrimal glands were histologically visible. Both the macroscopic aspect and the histological finding of sebaceous glands not typically found in the conjunctiva of the fornix, support this diagnosis. In addition, hair was found in the epidermal cysts and lead us to the assumption that these epidermal cysts could have grown in association with rather abnormal hair follicle development. Although the patient did not notice the supernumerary caruncle until the third decade of life, it can be assumed that it had existed since birth and was only discovered by the new onset of symptoms with foreign body sensation and itching. In the three cases of a supernumerary caruncle described in the literature, it was documented that the eye irritation was caused by surface irregularities and lanugo hairs; the symptoms disappeared after surgical excision.^6^^,^^10^^,^^11^

2. The **ectopic caruncle.** In contrast to the aforementioned supernumerary caruncle, a true ectopic caruncle is usually dysplastic and refers to a displacement of a tissue remnant from its normal commissural anatomic location, always at the medial posterior aspect of the lower eyelid. Such an anomaly may be associated with a number of other diseases of the ocular appendages. No case of ectopic caruncle was found in our case series.
3. **Megacaruncle** describes a considerable enlargement of the caruncle. In contrast to the supernumerary caruncle, there is a continuous connection of the additional caruncle tissue with the orthotopically located caruncle.^10^

In our second case with the diagnosis of a megacaruncle, a choristoma (tumor from non-local, normal tissue), but also true neoplasms of the caruncle can be considered as differential diagnosis. In a retrospective evaluation of 82 caruncle tumors at the University Eye Hospital Bonn between 1998 and 2020, one case of a caruncle choristoma, a dermoid, was described.^5^ No other dysgeneses of the caruncle were found. The most frequent differential diagnosis of a caruncle tumor was a caruncle nevus,^5^ which, however, is histologically out of the question in our case. In addition to sebaceous glands, our alteration includes fatty, connective and muscular tissues, which are present - albeit in much smaller proportions - in the normal caruncle as well. Therefore, we prefer the diagnosis of megacaruncle, which is ultimately a hamartoma. In case of unclear findings or complaints excision with subsequent histopathological examination is required. Histopathologically, structures that are typical for the caruncle but not the other parts of the conjunctiva like hair follicles and sebaceous glands can be found in caruncle dysgeneses, but not all of them are found in all cases of caruncle dysgeneses.

The oculo-auriculo-vertebral spectrum (OAVS) is a congenital disorder of craniofacial morphogenesis. It is characterized by malformations of structures derived from the first and second branchial arches, including the eyes, lips, tongue, palate, ear, maxilla and mandible. Goldenhar syndrome is part of the OAVS and describes mostly more severe manifestations.^12^ Caruncle malformations frequently occur in Goldenhar syndrome.^9^ Even if, as in this case series, malformations of the caruncle are also found in otherwise healthy patients, evidence for the presence of Goldenhar syndrome should be sought. Malformations of the face occur unilaterally in OAVS in many cases. In patient 3, a supernumerary caruncle was found only on the right side. This fits well with an increased occurrence on the right side of the facial malformations already described.^13^ In addition to the supernumerary caruncle, in Goldenhar syndrome the orthotopic caruncle may also have a bilobular shape or an ectopic caruncle may be present.^9^

Due to the association of malformations of the caruncle with Goldenhar syndrome, it is reasonable to assume that a disorder in the formation of the first and second branchial arches is responsible for the malformation even in otherwise healthy patients. Although the malformation of the caruncle is present from birth, it is usually not diagnosed until adulthood. Apart from cases in children with OAVS, only one case of a supernumerary caruncle in a 12-year-old child has been published so far.^8^ It is likely that hair follicles only develop sufficiently during and after puberty to cause symptoms that prompt patients to undergo ophthalmologic examination. Symptoms rarely occur overall. Therefore, it is possible that malformations of the caruncle (especially in children) are underdiagnosed. Due to the lack of function of the caruncle for the optic-visual system, insufficient attention may be paid to the caruncle to diagnose subtle changes.

## 4 Conclusions

In case of subjective complaints, size progression, changes or suspicion of malignancy, excision of the tissue with histopathological examination should always be performed. In most cases, the procedure can be performed under local anesthesia. Long-term surgical sequelae are not expected. Symptoms such as itching, foreign body and pressure sensation can be alleviated if the tumor is their cause. If the patient is symptom-free, the findings are stable in size, the slit-lamp microscopy is clear and there are no indications of malignancy, a “watch and wait” approach with regular ophthalmologic examinations may be considered.

## Patient consent

Written informed consent for publication of their clinical details and clinical images was obtained from the patients.

## Authors’ contributions

CJW and CAH contributed to the study conception and design. Material preparation, data collection and analysis were performed by CJW. The first draft of the manuscript was written by CJW and all authors commented on previous versions of the manuscript. All authors read and approved the final manuscript.

## Funding

We acknowledge support by the Open Access Publication Fund of the 10.13039/501100002714University of Freiburg.

## Data access, responsibility and analysis

CJW had full access to all the data in the study and takes responsibility for the integrity of the data and the accuracy of the data analysis.

## Availability of data and materials

The data presented in this study are available from the corresponding author upon reasonable request. The data are not publicly available due to privacy and ethical issues.

## Ethics approval and consent to participate

Approval was given by the local Ethics Committee of the University of Freiburg under the following number (22/15).

## Consent to publish

Written informed consent for publication of their clinical details and clinical images was obtained from the patients.

## Declaration of competing interest

The authors declare that they have no known competing financial interests or personal relationships that could have appeared to influence the work reported in this paper.

## Abbreviations

BE: both eyes
BCVA: best corrected visual acuity
HE: haematoxylin-eosin
IOP: intraocular pressure
LE: left eye
OAVS: oculo-auriculo-vertebral spectrum
RE: right eye
